# ^1^H NMR Spectroscopy and Multivariate Analysis of Monovarietal EVOOs as a Tool for Modulating Coratina-Based Blends

**DOI:** 10.3390/foods3020238

**Published:** 2014-04-17

**Authors:** Laura Del Coco, Sandra Angelica De Pascali, Francesco Paolo Fanizzi

**Affiliations:** Department of Biological and Environmental Sciences and Technologies (Di.S.Te.B.A.), University of Salento, Prov.le Lecce-Monteroni, Lecce 73100, Italy; E-Mails: laura.delcoco@unisalento.it (L.D.C.); sandra.depascali@unisalento.it (S.A.D.P.)

**Keywords:** olive oil, ^1^H NMR spectroscopy, monovarietal EVOO, PCA

## Abstract

Coratina cultivar-based olives are very common among 100% Italian extra virgin olive oils (EVOOs). Often, the very spicy character of this cultivar, mostly due to the high polyphenols concentration, requires blending with other “sweetener” oils. In this work, monovarietal EVOO samples from the Coratina cultivar (Apulia, Italy) were investigated and compared with monovarietal EVOO from native or recently introduced Apulian (Italy) cultivars (Ogliarola Garganica, Ogliarola Barese, Cima di Mola, Peranzana, Picholine), from Calabria (Italy) (Carolea and Rossanese) and from other Mediterranean countries, such as Spain (Picual) and Greece (Kalamata and Koroneiki) by ^1^H NMR spectroscopy and multivariate analysis (principal component analysis (PCA)). In this regard, NMR signals could allow a first qualitative evaluation of the chemical composition of EVOO and, in particular, of its minor component content (phenols and aldehydes), an intrinsic behavior of EVOO taste, related to the cultivar and geographical origins. Moreover, this study offers an opportunity to address blended EVOOs tastes by using oils from a specific region or country of origin.

## 1. Introduction

Extra virgin olive oil (EVOO) is undoubtedly an essential ingredient in the Mediterranean diet, and its beneficial effects on human health, such as the reduction of coronary heart disease risk factors, the prevention of several types of cancer and the modification of immune and inflammatory responses, are well known [1,2]. These benefits are mainly due to both the elevated oleic acid content and the antioxidant properties of its minor components [3], such as phytosterols, carotenoids, tocopherols and hydrophilic phenols. EVOO contains at least 30 phenolic compounds. The major phenolic compounds are oleuropein derivatives containing hydroxytyrosol, which are strong antioxidants and radical scavengers. Moreover, phenolic compounds are related to the sensory and nutritional qualities of EVOOs [4], which play an important role in the olive oil blending. In this regard, several investigations have been devoted to understand the correlations of phenolic compounds with the organoleptic properties (flavor, astringency and hardness) of foods [5] and to assess their content in olive oil. Recently, an experimental investigation was performed on blend and monocultivar EVOOs to estimate the perceived bitterness intensity by correlating sensory and chemical analysis (total phenol content spectrophotometrically measured) [6]. Other analytical methods, such as paper, thin-layer and column chromatography, as well as UV spectroscopy were applied to polyphenol analysis. Substantial developments have been realized by using high-resolution gas chromatography (GC) and high-performance liquid chromatography (HPLC) [4,7]. However, during the last decade, proton nuclear magnetic resonance spectroscopy (^1^H NMR) has been successfully employed in olive oil analysis [8]. The usefulness of ^1^H NMR spectroscopy has been increasingly known for its sample preparation ease, quickness and reasonable sensitivity to a wide range of compounds in a single measurement. Difficulties arise in relation to very low concentrated molecules detection and handling of all the information obtained from the spectra of multicomponent mixtures, such as olive oil [5]. In this regard, the multi-signal suppression sequence represents an interesting approach, since it exploits NMR signals not only of major lipid components (triglycerides), but also of minor compounds, without requiring any sample pretreatment, such as extraction and/or purification steps [9]. On the other hand, computer-based multivariate analyses software packages nowadays allow for the easy treatment of complex datasets coming from NMR spectra. In particular, ^1^H NMR spectroscopy coupled with chemometric studies has been used for the identification of EVOOs related to specific production areas and/or olive cultivars [10,11]. The absolute concentration and relative proportion of minor components are typical of each oil and may be used for production area and/or potential adulteration identification purposes. The fine composition of olive oil and, therefore, its sensory characteristics are influenced by several factors, such as climate, soil conditions, agricultural practices beside the specific cultivar used for its production [12,13]. In this work, we investigated by ^1^H NMR spectroscopy and multivariate analysis on monovarietal EVOO samples from different native or recently introduced Apulian (Italy) cultivars (Coratina, Ogliarola Garganica, Ogliarola Barese, Cima di Mola, Peranzana, Picholine), from Calabria (Italy) (Carolea and Rossanese) and from other Mediterranean countries, such as Spain (Picual) and Greece (Kalamata and Koroneiki). A pattern recognition method based on multivariate statistical analyses has been performed, by applying spectral bucketing rather than the integration of NMR signals. This approach provides first a global profiling overview of the data and allows one, thereafter, to focus only on the discriminant variables (buckets and then related metabolites signals) responsible for class differences, extracting the systematic variation simultaneously for all samples. Furthermore, variable selection may be used also in pattern recognition, in concert with domain knowledge, to select only biologically meaningful bucket regions of datasets for classification or dimensionality reduction [14]. The aim of this work was to offer an opportunity to address blended EVOO tastes, correlating the chemical composition and, in particular, the minor components (phenols and aldehydes) of monocultivar EVOOs with their use as potential taste smoothers and/or enhancers in the commercial blending preparation, using oils from a specific region or country of origin compliant with recent regional [15] or EU regulation [16]. Since EVOO is based on a growing business, where blends of different varietals of olive oils represent a high percentage of the market, the phenol and aldehyde NMR signals could allow the first qualitative evaluation of the characteristics for different monovarietal EVOO samples. This approach was also oriented towards the study of differences in polyphenol content among olive oil cultivars, their geographical origins and the suggestions for the blending of EVOOs for marketing purposes. Indeed, due to the strong taste, the EVOOs from the Coratina cultivar are less appreciated by some consumers [17]. Although the polyphenolic content is related to the bitterness and astringency of foods, it confers more oxidative stability and contributes to the sensory and nutraceutical quality of the oil. In this regard, we attempted to compare the EVOO Coratina samples with the other taste smoothing monocultivar EVOOs from Italy and/or other countries.

## 2. Experimental Section

### 2.1. Sample Collection

Monocultivar authentic EVOO samples were obtained during the harvesting period of 2012–2013, from Italy (Apulia and Calabria), Spain and Greece. In particular, monocultivar EVOO samples were: Coratina (10 samples from Bari, Apulia), Ogliarola Barese and Cima di Mola (10 samples for each cultivar, from Bari, Apulia), Ogliarola Garganica (10 samples from Gargano, Foggia, Apulia), Picholine (10 samples from north of Bari, Apulia) Peranzana (3 samples from San Severo, Foggia, Apulia), Carolea and Rossanese (8 and 3, respectively, from Calabria), Picual (3 samples from Spain) and Kalamata and Koroneiki (3 samples for each cultivar, from Greece) (Table 1). EVOOs were supplied from the company “Certified Origins Italia Srl” (Località Il Madonnino, Grosseto, Tuscany, Italy).

**Table 1:** Cultivar, the number of samples and the area of origin of the monocultivar authentic extra virgin olive oils (EVOOs) collected.

Cultivar (*cv.*)	Number of samples	Geographical origin
Coratina	10	Bari (Apulia, Italy)
Ogliarola Garganica	10	Gargano, Foggia (Apulia, Italy)
Ogliarola Barese	10	Bari (Apulia, Italy)
Cima di Mola	10	Bari (Apulia, Italy)
Picholine	10	Bari (Apulia, Italy)
Peranzana	3	San Severo, Foggia (Apulia, Italy)
Carolea	8	Calabria
Rossanese	3	Calabria
Picual	3	Spain
Kalamata	3	Greece
Koroneiki	3	Greece

### 2.2. Chemicals

All chemical reagents for analysis were of analytical grade. Chloroform-d (CDCl_3_, 99.8% atom D) and tetramethylsilane (TMS; 0.03% v/v) were purchased from Armar Chemicals (Switzerland).

### 2.3. NMR Spectroscopy

For NMR sample preparation, ~140 mg of olive oil were dissolved in deuterated chloroform (CDCl_3_ with TMS as the internal standard) adjusting the mass ratio of olive oil:CDCl_3_ to 13.5%:86.5%. Six hundred microliters of the prepared mixture were transferred into a 5-mm NMR tube. NMR spectra were recorded on a Bruker Avance III spectrometer (Bruker, Karlsruhe, Germany), operating at 400.13 MHz for ^1^H observation and a temperature of 300 K, equipped with a BBO (Broadband Observe) 5-mm direct detection probe incorporating a *z*-axis gradient coil. NMR spectra were acquired using Topspin 2.1 (Bruker). Automated tuning and matching, locking and shimming using the standard Bruker routines, ATMA (automatic tuning and matching in automatic mode), LOCK (frequency-field lock to offset the effect of the natural drift of the NMR’s magnetic field *B_0_*) and TopShim, were used to optimize the NMR conditions. Experiments were run in automation mode after loading individual samples on a Bruker Automatic Sample Changer interfaced with the software, IconNMR (Bruker). For each sample, after a 5-min waiting period for temperature equilibration, three ^1^H NMR experiments were performed: standard one-dimensional ^1^H ZG NMR experiment; one-dimensional ^1^H NOESYGPPS NMR pulse sequence; JRESGPPSGF with suppression of the strong lipid signals (20 frequencies), in order to enhance signals of the minor components present in EVOOs. Spectra were obtained by the following conditions: zg pulse program (for ^1^H ZGNMR), 64 K time domain, spectral width of 20.5555 ppm (8223.685 Hz), p1 (F1 channel—90° ^1^H transmitter pulse) 12.63 μs, pl1 −1.00 db (decibel), 16 repetitions; noesygpps1d.comp2 pulse program (for ^1^H NOESYGPPS NMR) 32 K time domain, spectral width 20.5555 ppm (8223.685 Hz), p1 12.63 μs, pl1 −1.00 db, 32 repetitions; jresgppsqf pulse program 8 K time domain, spectral width 16.7082 ppm (6684.492 Hz), pl1 −1 db, 4 repetitions for 40 experiments.

### 2.4. NMR Data Reduction and Preprocessing

NMR data were processed using Topspin 2.1 (Bruker) and visually inspected using Amix 3.9.13 (Bruker, Biospin). ^1^H NMR spectra were obtained by the Fourier transformation (FT) of the FID (free induction decay), applying an exponential multiplication with a line broadening factor of 0.3 Hz. The resulting ^1^H NMR spectra were manually phased and baseline corrected using the Bruker Topspin software. Chemical shifts were reported with respect to the TMS signal set at 0 ppm. ^1^H NMR spectra were segmented in rectangular buckets of a fixed 0.04-ppm width and integrated using the Bruker Amix software. Bucketing of the ^1^H NOESYGPPS NMR spectra was obtained within the range of 9.76–6.52 ppm. The spectral region between 7.60 and 6.92 ppm was discarded, because of the peak due to the residual protic chloroform signal at 7.24 ppm. The remaining buckets (64) were then normalized to the total area to minimize small differences and/or acquisition conditions among samples and, subsequently, mean-centered. The description of statistical analyses refers to unscaled data. The data table generated with all the spectra was submitted to multivariate data analysis.

### 2.5. Multivariate Statistical Analysis

Principal component analysis (PCA) was applied to NMR spectroscopic data. The multivariate statistical analysis and graphics were obtained using Simca-P version 13.0.2 (Umetrics, Sweden). PCA, an unsupervised pattern recognition method, was performed to examine the intrinsic variation in the dataset. The variables used for chemometric analyses were the buckets, which represent the portion of the NMR spectrum of phenolic components. Three separate PCA analyses describe the different performance of Coratina EVOOs with respect to monocultivar oils from various countries: Italy, Spain and Greece. Since the data is coming from different geographical scales (regional, inter-regional and national) and the limits defined by the principal axes were different, separate analyses were conducted. PCA was used to obtain a general overview of the NMR data and to describe the natural variability in the olive oils originating from specific cultivars. The *R*^2^ and *Q*^2^ are the two specific parameters considered for the description of the soundness of the models referring to the considered components. The former (*R*^2^) explains the total variation in the data, whereas the latter (*Q*^2^) is an internal cross-validation parameter, which indicates the predictability of the model. The predictive power of the model is estimated by determining how accurately we can internally predict the data, randomly removing some of them and verifying their correct classification. Therefore, a valid model consists of a good compromise between these two related parameters, *R*^2^ and *Q*^2^, indicating the explained variation and prediction goodness, respectively.

## 3. Results and Discussion—NMR Spectroscopy and Multivariate Statistical Analyses

The study of multi-suppression ^1^H NMR and the 2D *J-*resolved NMR spectra of monocultivar EVOO samples revealed a completely different profile in polyphenols contents. On the basis of the literature data [18,19], the identification of the main polyphenols was performed. As expected, monocultivar Coratina samples were characterized by intense NMR signals in the range at δ = 6.50–7.10 ppm, which could be assigned to phenyl alcohol moieties (tyrosol and hydroxytyrosol) of oleuropein and ligstroside and their aglycone. NMR signals at δ = 9.63 ppm were attributed to aldehydic forms of oleuropein and ligstroside; NMR signals at δ = 9.52 ppm and in the range of δ = 9.25–9.19 ppm were attributed to the dialdehydic forms of oleuropein and ligstroside [19]. It is well known that EVOO samples from *cv.* Coratina confer a bitter and astringent flavor to the oil. The bitter taste of olives is principally caused from oleuropein glucoside and its aglycone [18,20]. Then, oils obtained from olive fruits rich in polyphenols, e.g., cultivar Coratina, are expected to be more bitter and pungent. Recently Lauri *et al.* [18], in order to assess which chemical components are responsible for a given sensory descriptor, have looked for all possible correlations between the NMR signals (in the polyphenol spectral regions) and the analyzed sensory descriptors using OPLS (Orthogonal Partial least squares projections to latent structures) models. In particular, they explored the analytical potentiality of the NMR spectroscopy as a “magnetic tongue” in the analysis of EVOO, with particular attention to the quantitative measure of minor compounds related to the sensory description. Particularly, the phenol and aldehyde NMR signals allowed the first prediction of the sensory characteristics of EVOO.

**Figure 1:**
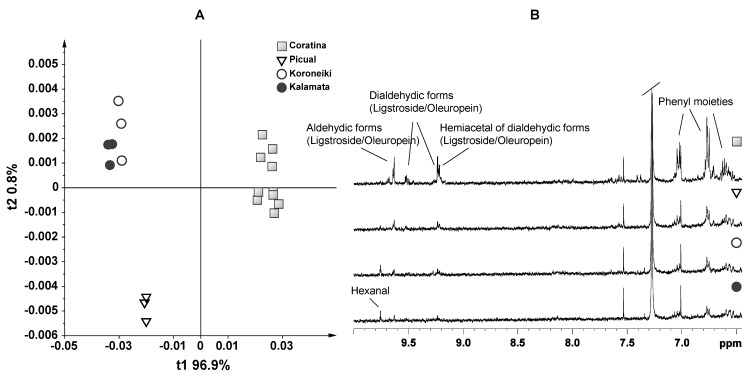
(**A**) PCA (t1/t2) score plot for monovarietal Coratina, Picual, Kalamata and Koroneiki samples (two components give *R*^2^ = 0.98 and *Q*^2^ = 0.943); (**B**) expansions of the ^1^H NMR spectra of monovarietal Coratina, Picual, Kalamata and Koroneiki samples used for the bucketing.

A further level of investigation was performed using the unsupervised exploratory statistical technique (PCA) on a bucket table made of 64 NMR buckets, from the polyphenol spectral region of 9.76–6.52 ppm. The bucket width was 0.04 ppm, and the spectra were all scaled to the total intensity before proceeding with the bucketing. PCA allowed the remapping the original dataset in a new multivariate coordinate space, where the dimensions are ordered by the decreasing explained variance in the data. The principal components were displayed as a set of scores (t), which highlight clustering or outliers, and a set of loadings (p), which highlight the influence of input variables on t. In all the models studied, PCA showed that by the first two components, a very useful model of the data was built. A first PCA was performed on EVOO samples of Coratina (Italy), Picual (Spain), Kalamata and Koroneiki (Greece), to obtain a general overview of the data grouping when Coratina was compared to cultivars of other countries (Figure 1). Thus, the first dimensions (principal components, t1/t2) highlight in a straightforward manner the spectra that were significantly different from each other and, ultimately, samples that showed distinct biochemical composition. Of the original 64 variables per spectrum, two components were enough to describe 98% of the variance of the entire NMR dataset, giving *R*^2^ = 0.98 and *Q*^2^ = 0.943. A good clustering of Coratina samples was observed along t1, whereas the Greek EVOO samples (Kalamata and Koroneiki, which were almost overlapped) were well separated from the Spanish EVOOs on the second component, t2. By analysis of the loading plot, Coratina samples showed a higher content of NMR signals at δ = 6.78, 9.22 and 9.62 ppm, which could be assigned to aldehydic and dialdehydic forms of oleuropein and ligstroside. These NMR signals in the recent work of Lauri *et al*. [18] have been correlated with bitter, pungent and artichoke tastes. The loading plot of t2 showed that the NMR Greek samples exhibited a higher concentration of signals at δ = 9.74, 9.50, 9.26 and 6.86 ppm. The signal at 9.74 ppm could be attributed to hexanal [21], molecule, associated with a sweet taste [22].

**Figure 2:**
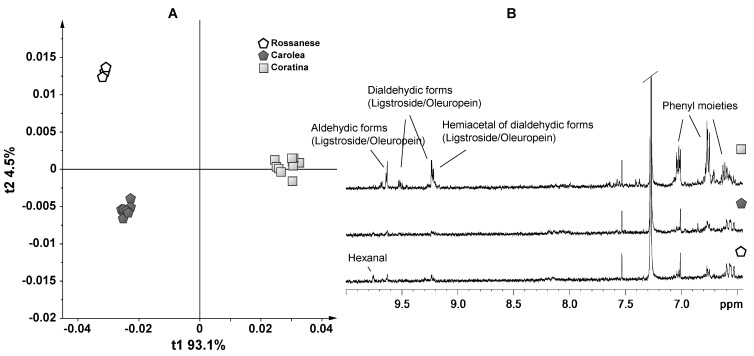
(**A**) PCA (t1/t2) score plot for monovarietal Coratina, Rossanese and Carolea samples (two components give *R*^2^ = 0.97 and *Q*^2^ = 0.95); (**B**) expansions region of the ^1^H NMR spectra of monovarietal Coratina, Rossanese and Carolea samples used for the bucketing.

A second PCA was performed on the Coratina and EVOO samples from Calabria (Figure 2). Also in this case, two components were able to describe 97% of the variance of the entire NMR dataset, giving *R*^2^ = 0.97 and *Q*^2^ = 0.95. Interestingly, there is a complete separation of the Coratina samples along t1 with respect to the Rossanese and Carolea, which, in turn, were split along t1. By examining the loadings of the original bucket variables, Coratina samples were characterized by variables with positive loadings on t1. These loadings, responsible for the marked clustering of Coratina, were again associated with the aldehydic and dialdehydic forms of oleuropein and ligstroside. Moreover, the Rossanese samples separated from the Carolea on the second component (t2), due to the positive loadings at 6.58 and 6.54 ppm, which could be assigned to phenyl alcohols moieties (tyrosol and hydroxytyrosol) of oleuropein and ligstroside aglycones [23]. The lack of aldehydic signals is usually related to a sweet taste.

**Figure 3:**
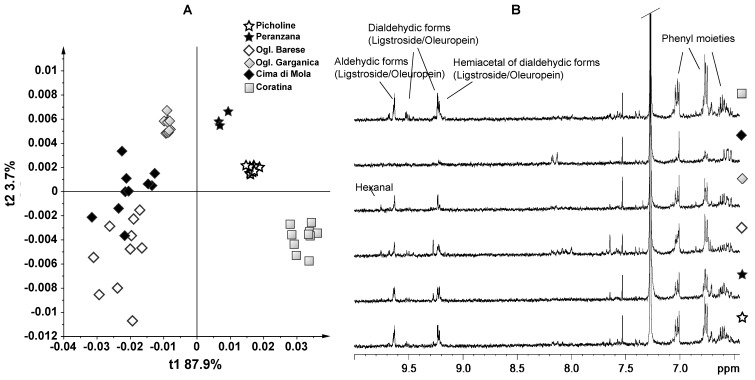
(**A**) PCA (t1/t2) score plot for monovarietal Apulian cultivar samples (native: Coratina, Ogliarola (Ogl.) Garganica, Ogliarola (Ogl.) Barese, Cima di Mola; recently introduced: Picholine and Peranzana). *R*^2^ = 0.95 and *Q*^2^ = 0.89; (**B**) expansions of the ^1^H NMR spectra of monovarietal Apulian cultivars samples used for the bucketing.

The last scatter plot was obtained by the PCA on all the EVOOs produced from Apulian cultivars (native: Coratina, Ogliarola Garganica, Ogliarola Barese, Cima di Mola; or recently introduced: Picholine and Peranzana) (Figure 3). This model built with six cultivars had a good descriptive ability with four components (t1 explains 87.9%, t2 3.7%, t3 2%, t4 1.1% of the total variance), which describe 95% of the variance with *R*^2^ = 0.95 and *Q*^2^ = 0.89. Looking at the score plot t1/t2, a good clustering of Coratina samples was observed. In particular, they were positioned at negative values of both t1 and t2, separating on t1 from the other native cultivar and along t2 with respect to the introduced cultivar. On the other hand, a certain degree of overlap was observed between two native cultivars, Ogliarola Barese and Cima di Mola, while Ogliarola Garganica was located at negative values of t1, but positive ones of t2. Finally, the recently introduced Picholine and Peranzana cultivars were clustered at positive values of both t1 and t2. Also in this case, a certain degree of variation in loadings levels was observed by the analysis of the loading plots. Along t1, the Coratina EVOOs showed again a higher content of molecules at δ = 6.78, 9.22 and 9.62 ppm, assigned to aldehydic and dialdehydic forms of oleuropein and ligstroside. The loading plot along t2 showed a clear separation of Coratina from both Picholine and Peranzana samples, due to a higher concentration for Coratina samples of buckets in the range from 6.74 to 6.78 ppm, from 9.18 to 9.22 ppm and at 9.50 ppm, which could be assigned to other aldehydic form of oleuropein [19]. Indeed, by comparison of the ^1^H NMR spectra of aromatic region of all native Apulian cultivars, a gradual decrement of aldehydic species from Coratina to Cima di Mola was observed. Interestingly, the contribution plot comparison (data not shown) of Ogliarola Barese and Ogliarola Garganica samples, which separated along t2 component, showed that the Ogliarola Barese EVOOs exhibited a higher concentration of molecules in the range of 7.94–8.10, 9.18, 9.26 and 9.50 ppm with respect to Ogliarola Garganica. In general, all these signals could be related to the rosemary flavor [18].

It should be noted that Coratina, Ogliarola Barese and Cima di Mola were the basic cultivars used for the Protected Designation of Origin (PDO) “Terra di Bari”, the most important PDO in the regional context and the second in Italy [24]. In all the studied cases, Coratina was the cultivar with the highest content of polyphenols, which could be related to the characteristic bitter and pungent taste. Among all the other cultivar considered, only the Picholine and Peranzana, cultivated in Apulia, showed a reasonable content in polyphenols. 

## 4. Conclusions

This study provides an initial evaluation of how natural variability in olive oil might affect blends originating from specific cultivars. Due to the importance of olive oil marketing for Italy and for other Mediterranean countries, it is very important to preserve its authentic characteristics. Since 90% of the entire oil production comes from Southern Italian Regions (Sicily, Calabria and Apulia), there is a real need to define the characteristics of local olive oil production. In particular, it should be noted that Coratina is the most popular olive cultivar of the Apulia region, among the leading olive oil producers in Italy, accounting for almost 40% of the total country production. As the Coratina cultivar has higher bitterness and very strong pungency, as declared by sensory analysis (panel test), the comparison of its molecular profile with other cultivars could be used to recognize the potential taste smoothers and/or enhancers in the commercial blending preparation. In this regard, NMR spectroscopy coupled with statistical multivariate analysis could be potentially applied to support and buttress taste analysis. All the studied samples were monocultivar EVOOs obtained from various Mediterranean countries (Greece, Spain) and Italian regions (Calabria, Apulia). The chemical profiles of the EVOO samples were analyzed by 1D and 2D ^1^H NMR spectroscopy, and assignment hypotheses were performed for NMR signals of the minor components of EVOOs. Different polyphenols were detected, especially in Coratina samples, even though no separation and/or isolation procedure of the phenolic fraction was conducted. According to the related loading plots, Coratina was the cultivar with the highest content of polyphenols, which could be related to the characteristic bitter and pungent taste. Among all the other cultivar considered, only the Picholine and Peranzana, cultivated in Apulia, showed a reasonable content of polyphenols. These signals were nearly absent in the oils commonly used to “smooth” the Coratina taste. Our intention was an explorative attempt to address the focused blending of monocultivar EVOOs being performed. The aim of the present work was therefore to give an indication of the possible taste smoother cultivar to be used rather than a specific blend production receipt. On the other hand, an example of a specific comparison for a blend with its monocultivar constituent EVOOs has been already recently reported [9]. This study also offers an opportunity to modify blended EVOO tastes by using oils from a specific region or country of origin compliant with recent regional [15] or EU regulation [16].
